# Mucosal defect closure after duodenal endoscopic submucosal dissection using the reopenable‐clip over the line method

**DOI:** 10.1002/jgh3.12577

**Published:** 2021-05-27

**Authors:** Tatsuma Nomura, Shinya Sugimoto, Nobuyuki Tsuda, Ryutaro Matsushima, Jun Oyamada, Akira Kamei

**Affiliations:** ^1^ Department of Gastroenterology Mie Prefectural Shima Hospital Shima Japan; ^2^ Department of Gastroenterology Ise Red Cross Hospital Ise Japan

## Abstract

**Introduction:**

Closure of mucosal defects after duodenal endoscopic submucosal dissection (ESD) is important to prevent postoperative adverse events. Previously, we devised an underwater reopenable‐clip closure method for effective closure of mucosal defects under endoscopic guidance within the field of view. Recently, the usefulness of a method using a clip with a line passing through an accessory channel to close a mucosal defect has been reported. We also described a reopenable‐clip over the line method (ROLM) to completely close margin and the muscular layers of mucosal defects using a clip line.

**Case Report:**

Our patient was a 70‐year‐old woman with a 40‐mm duodenal tumor in the descending portion of the duodenum. The lesion was completely resected using ESD . In the result, the mucosal defect size was approximately 50 mm, representing about 3/4 of the duodenal circumference. A clip‐line closure was performed using ROLM to close the mucosal defect's margins completely. An additional clip was applied to close the mucosal defect after ESD completely. Subsequently, the line was fixed with a modified locking‐clip technique, closed, and cut with endoscopic scissors. The patient was discharged without any adverse events 9 days after the duodenal ESD.

**Discussion:**

Mucosal defect closure after duodenal ESD using ROLM is a novel method that can reliably close mucosal defects.

## Graphical abstract

We described a reopenable‐clip over the line method to completely close margin and the muscular layers of duodenal mucosal defects using a reopenable‐clip and clip‐line.

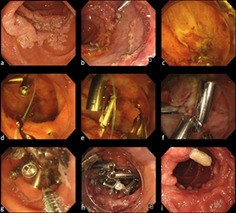

Closure of mucosal defects after duodenal endoscopic submucosal dissection (ESD) is important to prevent postoperative adverse events. Previously, we devised an underwater reopenable‐clip closure method for effective closure of mucosal defects under endoscopic guidance within the field of view.^1^ Recently, the usefulness of a method using a clip with a line passing through an accessory channel to close a mucosal defect has been reported.^2^ We also described a reopenable‐clip over the line method (ROLM) to completely close margin and the muscular layers of mucosal defects using a clip line.^3^ (Video S1).

ROLM uses a reopenable‐clip (Sure clip, Micro‐tech, Nanjing, China) and a line (0.23 mm nylon line). First, the line is fixed to the reopenable‐clip's teeth, and the clip with the fixed line is inserted through the accessory channel of the endoscope. The first clip grips the normal mucosa of the mucosal defect margins on the oral side and the muscular layer of the duodenal mucosal defect. Next, a second clip grips the normal mucosa on the anal side of the mucosal defect margin and the muscular layer of the duodenal mucosal defect. Finally, a line coming out of the accessory channel is passed through the tooth hole of the reopenable‐clip and inserted via the endoscopic accessory channel. The third clip is inserted via the endoscopic accessory channel using ROLM and is placed such that the mucosal defect margins on the oral and anal sides are fixed. In underwater conditions, mucosal defect closure after duodenal ESD causes softening of the duodenal muscle layer. Also, if the clip unexpectedly grasps the mucosa and muscle layer at an unintended site, it can easily be reopened to regrasp the mucosa and muscle layer at the correct location.

Our patient was a 70‐year‐old woman with a 40‐mm duodenal tumor in the descending portion of the duodenum (Fig. 1). The tumor was pointed out by esophagogastroduodenoscopy during a medical check‐up and it was located on the anal side of the papilla of Vater. The lesion was completely resected using ESD under conscious sedation with intravenous anesthesia by using midazolam and pentazocine. In the result, the mucosal defect size was approximately 50 mm, representing about three‐fourths of the duodenal circumference. A clip‐line closure was performed using ROLM to close the mucosal defect's margins completely. An additional clip was applied to close the mucosal defect after ESD completely. Subsequently, the line was fixed with a modified locking‐clip technique, closed, and cut with endoscopic scissors (Loop Cutter FS‐5U‐1; Olympus Medical, Tokyo, Japan).^4^ One day later, X‐ray radiography was used to confirm that almost all clips were still in place. The patient was managed with fasting for 6 days after the duodenal mucosal defect was closed, and proton pump inhibitors were administered. The patient was discharged without any adverse events 9 days after the duodenal ESD.

**Figure 1:**
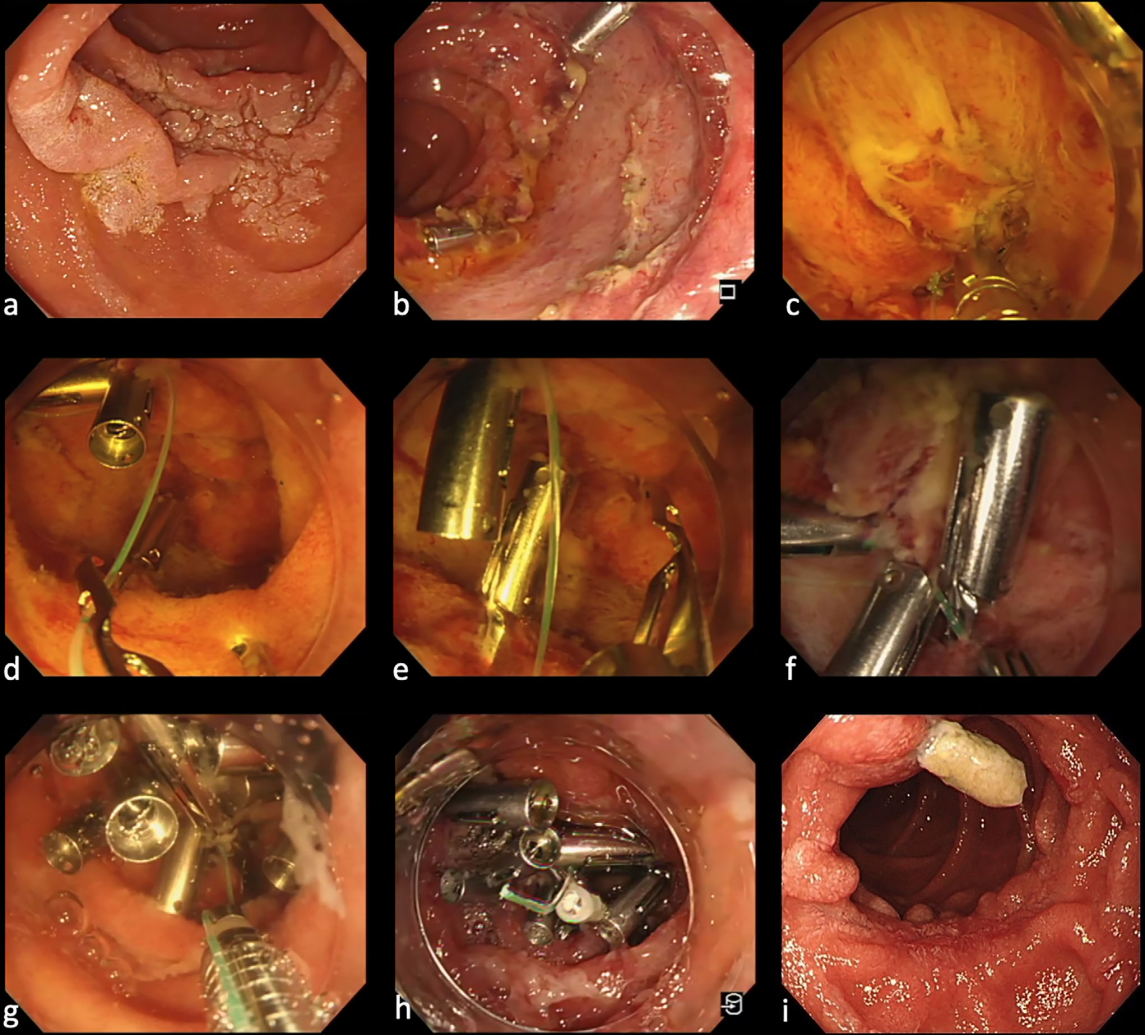
(a) Paris, type 0‐IIa lesion of the descending portion of the duodenum. (b) Mucosal defect after duodenal ESD. (c) Place a reopenable‐clip fixed line on the oral mucosal defect. Next, a second clip grips line, the normal mucosa on the anal side of the mucosal defect margin and the muscular layer of the duodenal mucosal defect. (d) Using the reopenable‐clip over the line method (ROLM), a reopenable‐clip with a line passed through the tooth appears on the endoscope view. (e, f) By pulling the line slightly, the mucosal defect's margins are brought close to each other, and the mucosal defect's margins are fixed firmly. (g) Fixing the line with the modified locking‐clip technique before cutting to prevent it from slipping off the clip. (h) Completely closed mucosal defect. (i) The endoscopic image of the duodenal mucosal defect at the follow‐up after 2 months.

Mucosal defect closure after duodenal ESD using ROLM is a novel method that can reliably close mucosal defects.

## Supporting information


**Video S1.** Actual mucosal defect closure using ROLM for mucosal defect after duodenal endoscopic submucosal dissection. Large size video file can be accessed at https://www.dropbox.com/s/xepjk4k2pupjvbe/ROLM%20JGH%20Large%20size%20.m4v?dl=0.
